# Posture-Specific Breathing Detection

**DOI:** 10.3390/s18124443

**Published:** 2018-12-15

**Authors:** Hualin Guan, Xiaodong Yang, Wanrong Sun, Aifeng Ren, Dou Fan, Nan Zhao, Lei Guan, Daniyal Haider, Qammer H. Abbasi

**Affiliations:** 1 School of Electronic Engineering, Xidian University, Xi’an 710071, China; guanhualin1993@163.com (H.G.); sunwanrong@xidian.edu.cn (W.S.); afren@mail.xidian.edu.cn (A.R.); dfan9308@gmail.com (D.F.); nan_zhao_@hotmail.com (N.Z.); daniyalhaider86@gmail.com (D.H.); 2 School of Life Sciences and Technology, Xidian University, Xi’an 710126, China; 15926395470@163.com; 3 School of Engineering, University of Glasgow, Glasgow G12 8QQ, UK; Qammer.Abbasi@glasgow.ac.uk

**Keywords:** non-invasive, respiratory activity detection, body postures

## Abstract

Human respiratory activity parameters are important indicators of vital signs. Most respiratory activity detection methods are naïve abd simple and use invasive detection technology. Non-invasive breathing detection methods are the solution to these limitations. In this research, we propose a non-invasive breathing activity detection method based on C-band sensing. Traditional non-invasive detection methods require special hardware facilities that cannot be used in ordinary environments. Based on this, a multi-input, multi-output orthogonal frequency division multiplexing (MIMO-OFDM) system based on 802.11n protocol is proposed in this paper. Our system improves the traditional data processing method and has stronger robustness and lower bit relative error. The system detects the respiratory activity of different body postures, captures and analyses the information, and determines the influence of different body postures on human respiratory activity.

## 1. Introduction

The human body respiratory system is sensitive and respiratory activity parameters of the human body are important indicators of vital signs. Respiratory activity indicates the basic health and state of the living body. Abnormalities in respiratory activity parameters are often accompanied by medical emergencies [1,2,3]. Therefore, detecting respiratory activity plays an important role in many situations. 

In the healthcare sector, invasive respiratory monitoring methods are easy and safe but have many limitations. These invasive methods force the user to wear electrodes or cables for sensing, which limits their capability to sense during special occasions (such as post-earthquake life information detection) and have a limited application range. The electrodes cause major interference in the measurement of physiological parameters, affecting the accuracy of respiratory detection. Many operations, such as electrode placement, which is difficult to avoid in contact measurement, requires the user to actively participate in the test, and in some cases, the user may not meet this requirement [2].

Non-contact (non-invasive) respiratory activity monitoring is the solution to these issues. Non-contact respiration detection technology measures the respiratory parameters of living organisms without touching the living body to not only provides a non-invasive, convenient, and more suitable detection means for respiratory activity detection, but also to provide the possibility of detecting physiological signal features in special occasions [4].

Non-contact respiratory activity detection methods include infrared, video, electrostatic field, gas composition, ultrasonic waves, and electromagnetic waves. Ultrasonic and electromagnetic waves use the Doppler principle to retrieve non-contact signal information, which detects the existence of and provides information about the respiratory activities of living bodies [5,6,7]. Electromagnetic waves have unique advantages in the detection of physiological signals, and are not susceptible to environmental factors such as weather, temperature, and light, with strong penetrating power [6]. 

Because of its non-invasive and robustness to low-light sleep environments, radio frequency (RF) technology has attracted wide attention as a potential candidate technology for tracking human breathing. The basic principle behind this method is that exhalation and inhalation cause minor changes in the transmission of radio signals from the transmitter (TX) to the receiver (RX). In this category, some systems use special wireless devices such as Doppler radar [8], UWB MIMO (Ultra-wideband multi-input, multi-output) radar [9], USRP (universal software radio peripherals) [10], or FMCW (frequency modulated carrier waves) [11,12]. However, these systems rely on special hardware devices, limiting their practicality in many applications.

In this paper, a new method of C-band sensing technology based on 802.11n protocol is adopted in a MIMO-OFDM (multi-input, multi-output orthogonal frequency division multiplexing) system, which includes multiple transmitting antennas for AP (access point), multiple receiving antennas for the network card, and carrier modulation for OFDM technology, working at 5.32 GHz. Compared with a single TX-RX pair, this system has lower relative error and better robustness.

The main idea of this paper is as follows. The respiratory activity of the human body, based on C-Band sensing, was tested to verify the effectiveness of the system. The proposed system improves upon traditional breathing data filtering methods, having better robustness and lower relative error. The improved system was used to detect the human respiratory activity under different postures. As different postures influence human respiratory activity, we found that standing has the greatest influence on respiratory activity.

## 2. System Design

### 2.1. System Flow

**Figure 1:**
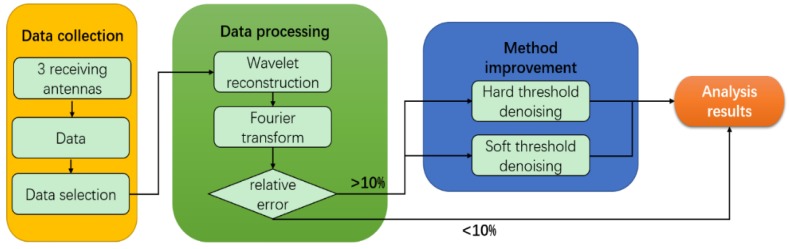
The logic flow of our system.

Figure 1 depicts the process of the proposed system. The first part is data acquisition, which involves collecting signal data through three receiving antennas. These data can be transmitted or stored locally in real time, and then the characteristics of the data are selected to find the most suitable raw data for processing (detailed in Section 3.1). The second part is data processing. This part mainly involves preliminary analysis of the collected data to determine whether the results meet the requirements. When the relative error between the frequency after data processing and the reference frequency is more than 10%, the third stage is required; otherwise, this stage is skipped. Finally, the fourth stage involves improving and preserving the results.

### 2.2. Data Collection

**Figure 2:**
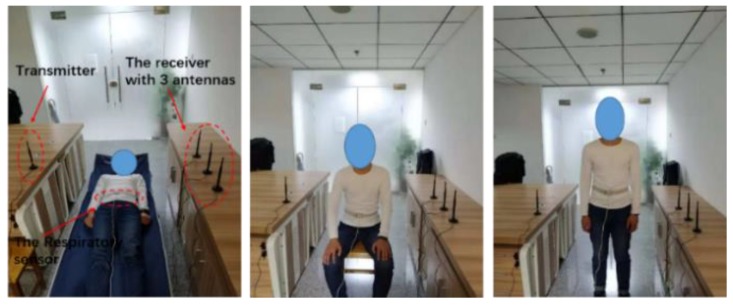
C-band sensing-based human respiratory activity information detection model.

Figure 2 shows the device and the experimental environment used for obtaining wireless data. We mainly collected three kinds of breathing data from the subjects: lying down, sitting, and standing. As shown in Figure 2, we implemented the experiment in a large room (5 × 8 m^2^).

Data acquisition is divided into two parts: the acquisition of electromagnetic wave signal and the collection of respiratory sensor signals. For respiratory sensor data collection, as shown in Figure 2, the respiratory sensor is tied to the abdomen of the subjects. This device is an HKH-11C Digital Respiratory Sensor (Electronic Technology Research Institute, Hefei, China). The sampling frequency of this sensor is 50 Hz, data are 8 bits, and baud rate is 9600. The sensor is compact and safe to transport. For the acquisition of the electromagnetic wave signal, the transmitter antenna is deployed as an access point (AP) that operates at 5.32 GHz. The receiving antennas are placed opposite the AP, 1.5 m apart. The subject is in the middle of the transmitter and the receiver, as shown in Figure 2. The sample rate was set to 100 packets/s. When conducting experiments, we obtained a certain number of packets. Each channel state information (CSI) packet’s amplitude and phase information are related as follows:

(1) $$H_{i}=\left|H_{i}\right|e^{-i∠H_{i}},$$

where $\left|H_{i}\right|$ represents the CSI amplitude of the *i*th subcarrier and $∠H_{i}$ represents the CSI phase of the ith subcarrier.

In this experiment, the 5.32 GHz C-band signal was adopted based on the IEEE 802.11 n standard. C-Band sensing describes the physical properties of the channel between transceivers in an OFDM wireless communication system, which is the physical layer level, and can provide channel measurements at the subcarrier level [13]. C-Band sensing describes the characteristics of the channel in the form of data packets. The path loss, multipath propagation, and distortion experienced by the signal when propagating the original data are reflected in C-Band sensing. Commercial wireless network cards that currently support the IEEE 802.11 n standard can provide amplitude and phase information for different carriers in the form of a C-Band sensing channel matrix.

In a narrow bandwidth stationary channel, the OFDM system can be simply modeled in the frequency domain as:

(2) Y=HX+N ,

where Y is the received vector, X is the transmitted vector, H is the channel matrix, and N is the Additive White Gaussian Noise (AWGN).

Therefore, we can obtain the channel state transition matrix:

(3) $$Y=\left[\begin{array}{c} y_{1}\\ y_{2}\\ \vdots \\ y_{n} \end{array}\right]\;X=\left[\begin{array}{c} x_{1}\\ x_{2}\\ \vdots \\ x_{m} \end{array}\right]\;H=\left[\begin{array}{ccc} h_{11} & \begin{array}{cc} h_{12} & \cdots \end{array} & h_{1m}\\ \begin{array}{c} h_{21}\\ \vdots \end{array} & \begin{array}{cc} h_{22} & \cdots \\ \vdots & \ddots \end{array} & \begin{array}{c} h_{2m}\\ \vdots \end{array}\\ h_{n1} & \begin{array}{cc} h_{n2} & \cdots \end{array} & h_{nm} \end{array}\right]\;N=\left[\begin{array}{c} n_{1}\\ n_{2}\\ \vdots \\ n_{n} \end{array}\right]$$

where *m* is the number of transmitting antennas and *n* is the number of receiving antennas. In this experiment, *m* equals 1 and *n* equals 3.

### 2.3. Data Processing

The purpose of data processing is to denoise the original data representing the Y matrix using several common methods for electromagnetic wave denoising to determine whether the results meet the expected conditions. Previous studies [14,15,16,17,18] showed that, among the several traditional methods, wavelet transform has the best filtering effect. Therefore, in this process, wavelet transform was chosen.

### 2.4. Method Improvement

According to the previous research results, we found many optimization methods for denoising. In this paper, we chose relatively simple and effective methods: hard threshold denoising and soft threshold denoising.

### 2.5. Analysis Results

After processing the data, we analyzed it if we obtained the expected result. Here, we mainly analyzed the effects of different postures on respiration.

## 3. Processing of C-Band Sensing

### 3.1. Data Pre-Processing

**Figure 3:**
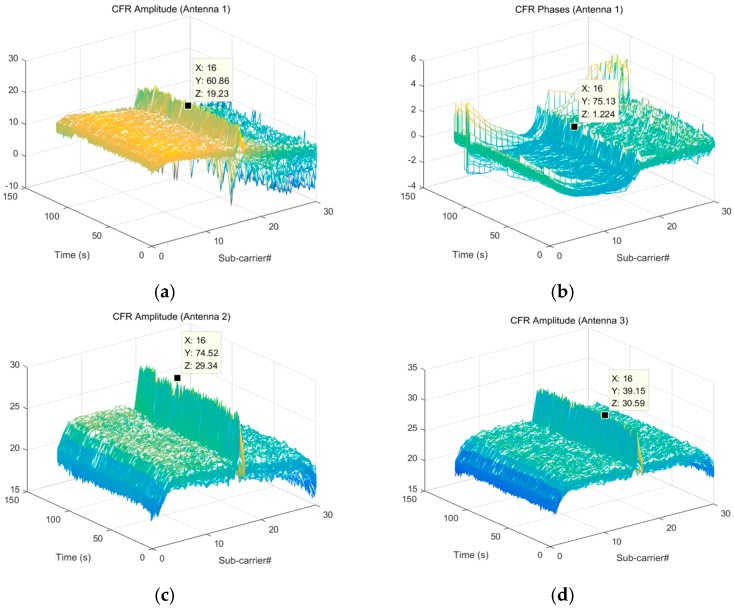
(**a**) The CFR (channel frequency response) amplitude from antenna 1. (**b**) The CFR phase from antenna 1. The CFR amplitude from (**c**) antenna 2 and (**d**) antenna 3.

When a person lies down, we measured the electromagnetic wave signal using the C-band sensing system. Figure 3 shows the data collected by the three receiving antennas, each containing 30 subcarrier signals of channel frequency response (CFR). In order to obtain the correct respiratory activity data, we had to find the best single subcarrier. The CFR includes amplitude/frequency response and phase/frequency response. In this experiment, the amplitude/frequency response was can be used to intuitively analyze the time-frequency characteristics of a signal, so the this was used to process the respiratory signal. As can be seen from Figure 3, the 16th subcarrier of all three antennas had the most obvious signal characteristics. In order to further determine which subcarrier to choose, we made a preliminary judgment by calculating the standard deviation.

**Figure 4:**
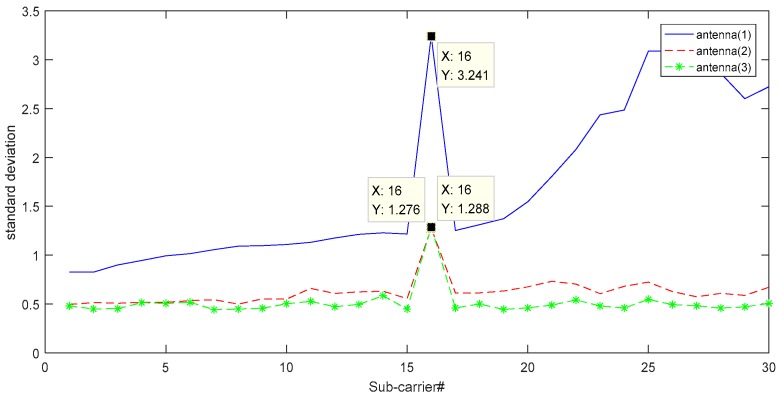
Standard deviation of the subcarriers of the three antennas.

Figure 4 shows the standard deviation of the subcarriers of the three antennas. The more the subcarrier is affected by the respiratory signal, the greater the standard deviation of the modulated signal. Therefore, the subcarrier with the largest standard deviation most easily obtains the respiratory signal. From Figure 4, we found that all three antennas have the largest standard deviation for the 16th subcarrier. Among the three antennas, the signal received by antenna 1 was unstable, and the standard deviation of the 16th subcarrier of antenna 3 was the largest. In summary, the 16th subcarrier of the third antenna was selected in this experiment.

The data collected in this section are the respiratory data of healthy adults while lying down. There were 10 groups of data: each set of data included 2 min of acquisition time, and the sampling frequency was 100 Hz. In each group of data acquisition, the tester was connected to the respiration sensor, and data accuracy was verified by comparing the data of the respiration sensor with the processed data.

The relative error was calculated as follows:

(4) $$E=\frac{\left|f_{s}-f_{t}\right|}{f_{s}}\times 100\%$$

where E is the relative error, $f_{s}$ is the human respiratory frequency measured by the respiratory sensor, and $f_{t}$ is the human respiratory frequency after C-Band sensing data processing.

**Figure 5:**
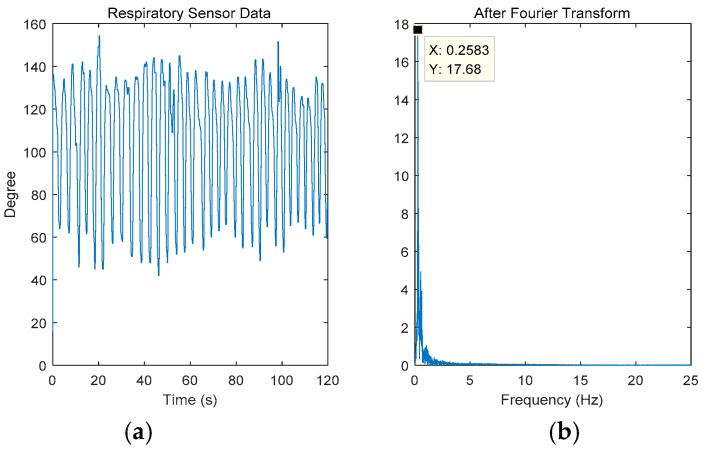
The respiratory signal measured by the respiratory sensor when laying down: (**a**) the time domain signal and (**b**) the frequency domain signal after Fourier transform.

Figure 5 shows the data measured by the respiratory sensor corresponding to this set of data. Because the data obtained by the breathing sensor were stable and experienced little interference, the breathing frequency was obtained by directly performing Fourier transform. The sampling frequency of the breathing sensor was 50 Hz, the sampling time was 2 min, and the total number of samples was 6000. Given this information, correct Fourier transform could be applied. The ordinate in Figure 5a,b shows the degree of undulation of the abdomen rather than the amplitude. As shown in the figure, the frequency was 0.2583 Hz with respiration rate of 15–16 bpm (beat per min). Figure 5b shows the Fourier transform of Figure 5a.

### 3.2. Wavelet Transform

In order to obtain breathing signals from the 16th subcarrier of the third antenna, the noise contained in CFR data had to be eliminated. We considered it inappropriate to use traditional filters (e.g., the Butterworth and Chebyshev filters) to remove the high frequency noise contained in CFR because they an not only eliminate noise but also blur the possible rise and fall edges of CFR signals, which are essential for detecting sleep apnea and rollover. Here, we applied the wavelet filter proposed in (Demeechai, Kukieattikool, et al.) [18], because it retains the sharp conversion of signals better than other low-pass filters. More specifically, we applied the 8-level b3 wavelet transform to each CFR sequence, and only used detailed coefficients to re-construct. Since the sampling frequency of the data was 100 Hz, the frequency range of the signal obtained was 0–50 Hz. After 8-level wavelet decomposition, the frequency range of detail coefficient was about 0.19–0.38 Hz, which includes the normal range of human respiratory frequency.

**Figure 6:**
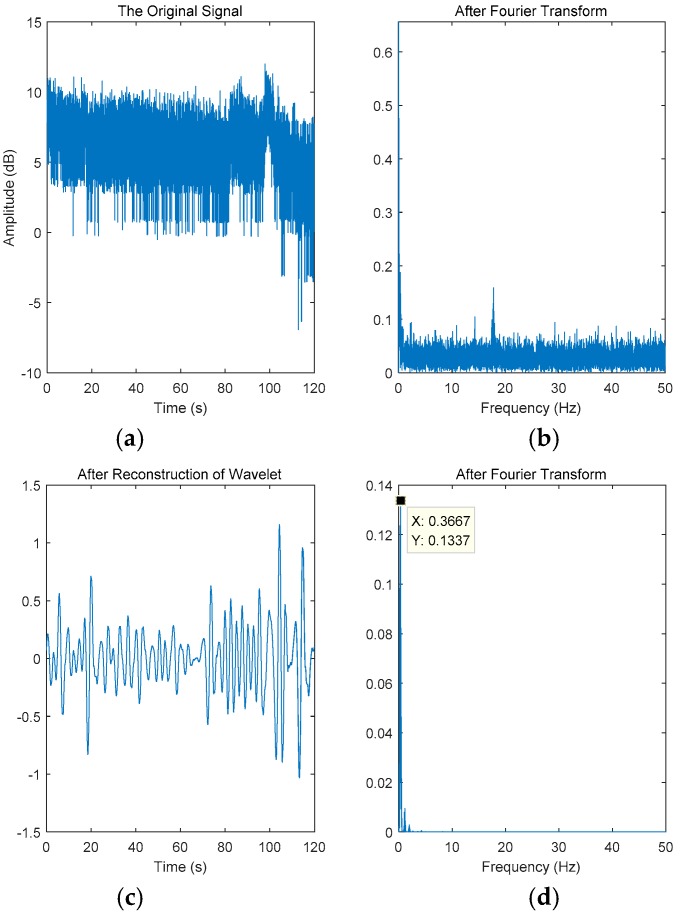
The data of the 16th subcarrier of the third antenna for a person laying down. (**a**) Time domain signal of the subcarrier. (**b**) Fourier transform of subcarrier. (**c**) Time domain signal after subcarrier wavelet reconstruction. (**d**) Fourier transform of wavelet reconstruction signal.

Figure 6 shows the subcarrier signal in the C-band sensing data. Figure 6a,b are the original subcarrier signals. Among them, the ordinate unit in Figure 6a is dB. Figure 6c is a signal reconstructed from the eighth-order wavelet detail coefficients and Figure 6d depicts the signal obtained after the Fourier transform of the reconstructed signal. The purpose of Fourier transform is to acquire the frequency domain characteristics of the signal, to compare with the frequency domain characteristics of the breathing sensor, and to obtain the relative error (E). As shown in Figure 6b, the frequency of the original C-band sensing data was almost zero, and the other part was uniformly distributed in the range of 0 to 50 Hz. After the filtration of C-band sensing data, the measured respiratory signal frequency was 0.3667 Hz and the relative error was 42%.

This relative error was still high, and an improved algorithm was needed to reduce the relative error. This method needed to have good robustness and good performance for different experimenters.

### 3.3. Improvements to Wavelet Transform

The wavelet threshold shrinkage method was proposed by Donoho and Johnstone in 1995. Threshold denoising, in short, decomposes the signal, then performs threshold processing on the decomposed coefficients, and finally reconstructs the denoised signal. Wavelet transform has strong de-data correlation, which can concentrate the energy of the signal in some small wavelet coefficients in the wavelet domain, whereas the energy of the noise is distributed in the whole wavelet domain. Therefore, after wavelet decomposition, the amplitude of the wavelet coefficient of the signal is greater than the amplitude of the coefficient of the noise. The wavelet coefficients with larger amplitudes are generally dominated by signals, whereas the smaller amplitude coefficients are considered noise. Thus, the threshold can be used to preserve the signal coefficients and reduce most of the noise to zero [19,20,21]. The specific process of wavelet threshold shrinkage denoising is: wavelet decomposition of the noisy signal on each scale, and setting a threshold. Then, the wavelet coefficient whose amplitude is lower than the threshold is set to 0, and the wavelet coefficient is higher than the threshold or completely reserved, or perform the corresponding shrinkage processing. Finally, the wavelet coefficients obtained after processing are reconstructed by inverse wavelet transform to obtain the denoised signal.

In the process of wavelet decomposition threshold denoising, the threshold reflects the different processing strategies for wavelet coefficients, exceeding and below the threshold, which is a key step in threshold denoising. Let W denote the wavelet coefficient, T is the given threshold, and sign(*) is the symbol function. Common threshold functions are the hard and soft threshold functions.

In the hard threshold function, the coefficient of the absolute value of the wavelet coefficient below the threshold becomes zero, and the coefficient above the threshold remains unchanged:

(5) $$W_{new}=\{\begin{array}{c} 0\;\;;\left|W\right|<T\\ W\;;\;\left|W\right|>T \end{array},$$

In the soft threshold function, the coefficient of the absolute value of the wavelet coefficient below the threshold becomes zero, and the coefficient above the threshold shrinks:

(6) $$W_{new}=\{\begin{array}{c} 0\;\;\;\;\;\;\;;\left|W\right|<T\\ sgn\left(W\right)\left(\left|W\right|-T\right);\left|W\right|>T \end{array},$$

Notably, (1) the hard threshold function is discontinuous at the threshold point, and discontinuity will cause ringing and the pseudo-Gibbs effect. (2) With the soft threshold function, there is always a constant deviation of the original coefficient and the wavelet coefficient obtained by decomposition, which will affect the accuracy of reconstruction.

The best method is to select a threshold above the maximum noise level. It was proven that the value of noise is lower than $\sigma \sqrt{\log_{e}Length}$ (proposed by Donoho) with a very high probability, where the parameter to the right of the root (called “sigma”) is the estimated noise standard deviation [19,20,21]. σ represents the standard deviation of the wavelet coefficients and Length represents the length of the original subcarrier signal. Here, we used $\sigma \sqrt{\log_{e}Length}$ as a threshold, $T=\sigma \sqrt{\log_{e}Length}$.

In the previous section, the eighth-order wavelet decomposition was performed, and then the detail coefficients were reconstructed, resulting in a high relative error. In this experiment, σ equals the standard deviation of the eighth-order wavelet detail coefficient and Length  equals the length of the original signal, which was 12,000.

**Figure 7:**
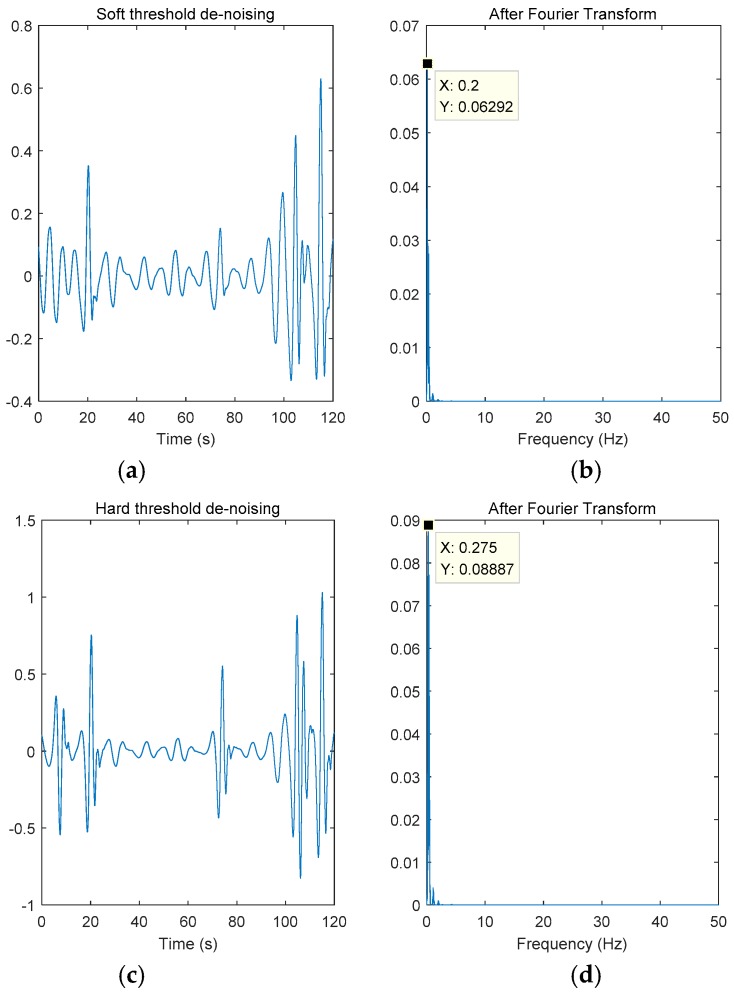
Human respiratory signal after threshold denoising. (**a**) Time domain signal and (**b**) Fourier transform after soft threshold denoising. (**c**) Time domain signal and (**d**) Fourier transform after hard threshold denoising.

From Figure 7b,d, we can see that the respiratory activity frequency after soft threshold denoising was 0.2 Hz and the respiratory activity frequency after hard threshold denoising was 0.275 Hz. After calculating the relative error, we concluded that the soft threshold denoising relative error was 22.6% and the hard threshold denoising relative error was 6.5%.

However, this is only the result for one set of data, which may be accidental. Therefore, all 10 sets of data were processed similarly. 

**Figure 8:**
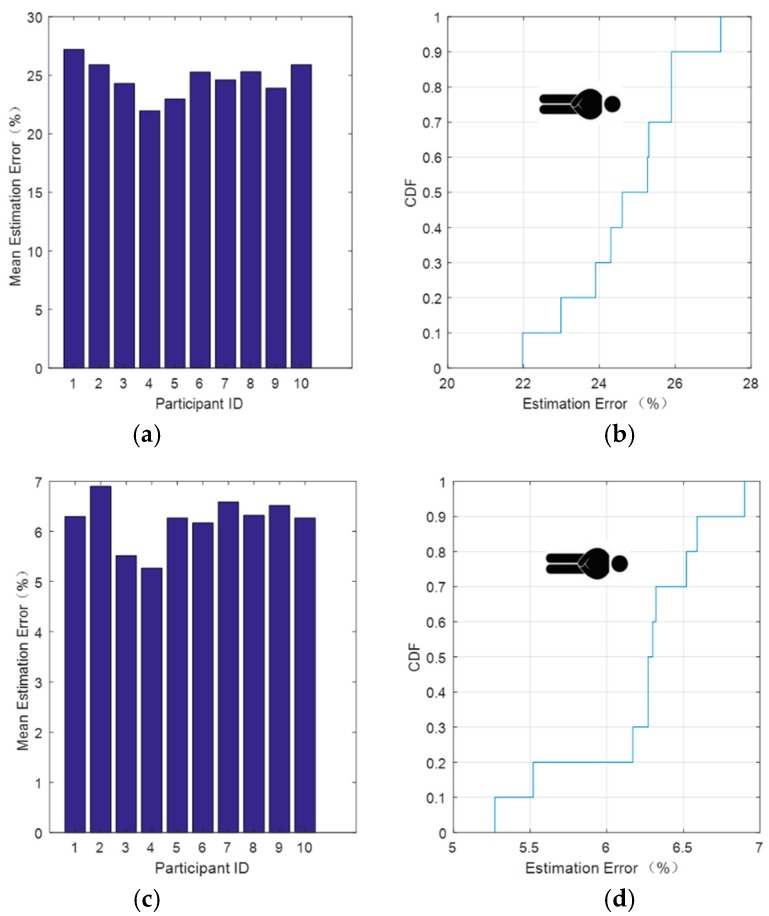
Respiratory signal relative error after data processing while lying down. (**a**) Average error estimate and (**b**) CDF (cumulative distribution function) of the estimated error after soft threshold denoising. (**c**) Average error estimate and (**d**) CDF of the estimated error after hard threshold denoising.

Figure 8 shows the resulting relative error and its cumulative distribution function (CDF) after the improved method was used to process the 10 sets of data.

From Figure 8, we can see that after threshold denoising of the wavelet reconstructed signal, the relative error significantly decreased. Figure 8b shows that, after soft threshold denoising, there was a 90% probability that the relative error would be lower than 26% and a 100% probability that it would be less than 28%. Figure 8d shows that, after hard threshold denoising, there was a 80% probability that the relative error would lower than 6.5% and a 100% probability that it would be less than 7%. This method was also applied to each group of data. Even if the experimenter was different, there was no need to modify the parameters of the processing method, as it demonstrated good robustness.

Through these 10 sets of data, we concluded that the healthy adult test subject has a breathing frequency of about 0.25 Hz when lying down with breathing rate of 15 bpm, under normal circumstances.

## 4. Research on Respiratory Activity Detection under Different Postures

### 4.1. Information Processing of Respiratory Activity in Different Postures

The human body has different postures at different times, which produce different respiratory activity. However, due to the diversity and complexity of human behavior, we collected the breathing information of several representative body postures as typical body breathing activity information.

**Figure 9:**
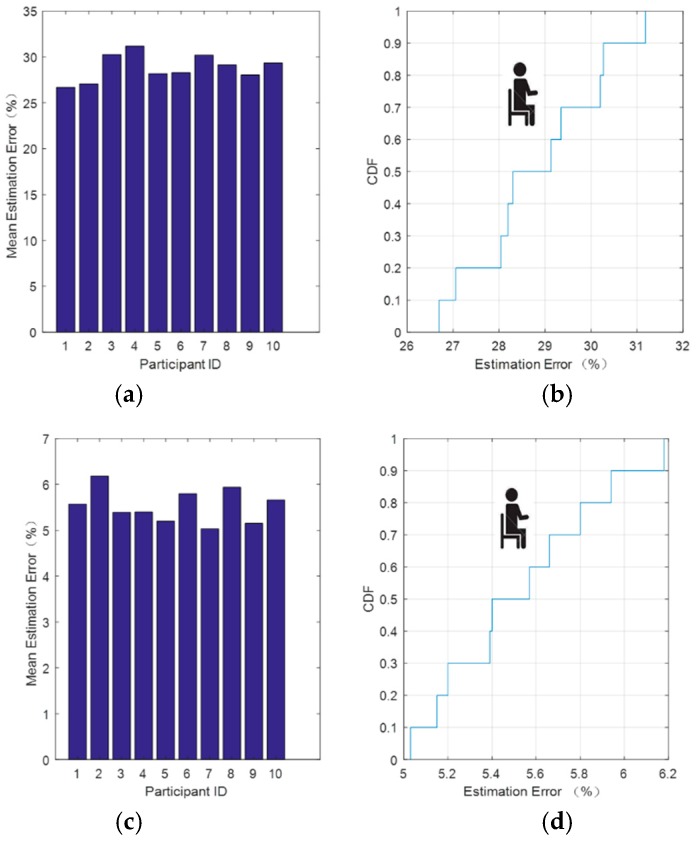
Respiratory signal relative error after data processing for the sitting posture. (**a**) Average error estimate and (**b**) CDF of the estimated error after soft threshold denoising. (**c**) Average error estimate and (**d**) CDF of the estimated error after hard threshold denoising.

**Figure 10:**
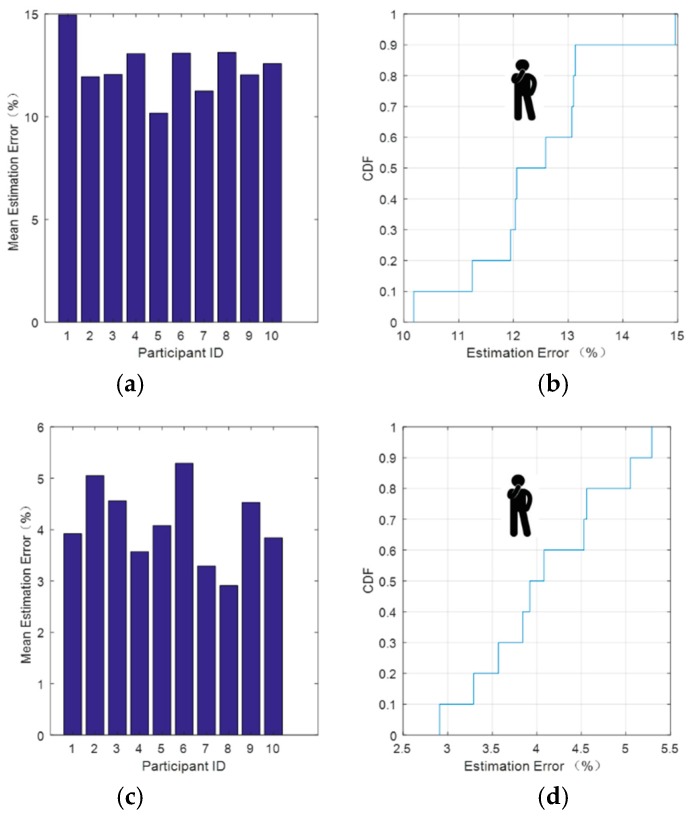
Respiratory signal relative error after data processing for the standing position. (**a**) Average error estimate and (**b**) CDF of the estimated error after soft threshold denoising. (**c**) Average error estimate and (**d**) CDF of the estimated error after hard threshold denoising.

We used the improved method outlined in the previous section to process the C-band sensing data while sitting and standing. We found that the relative error of the hard threshold denoising remained low and performed well as shown in Figure 9 and Figure 10.

Figure 9 shows the respiratory signal relative error after data processing for the sitting posture. Figure 9b shows that after soft threshold denoising, there was a 70% probability that the relative error would be lower than 30% and a 100% probability that it would be less than 32%. From Figure 9d, we can see that after hard threshold denoising, there was an 80% probability that the relative error would be lower than 5.8% and 100% probability that the relative error would be less than 6.2%. This method is also applicable to each group of data as mentioned earlier.

Figure 10 depicts the respiratory signal relative error after data processing for the standing position. It can be seen from Figure 10b that after soft threshold denoising, there was a 60% probability that the relative error would be lower than 13% and a 100% probability that it would be less than 14%. From Figure 10d, after hard threshold denoising, there was an 80% probability that the relative error would be lower than 5% and 100% probability that it would be less than 5.5%. This method is also applicable to each group of data and behaves same as mentioned above.

According to the measured data, the breathing frequency of the subject while sitting was 0.3 Hz, with breathing rate of 18 bpm; the subject’s breathing frequency while standing was 0.333 Hz, with respiration rate of 20 bpm.

### 4.2. Comparative Analysis of Respiratory Activity Detection for Different Postures

Table 1 was created by combining the lying down, sitting, and standing data.

From Table 1, the improved data processing method has low relative error and maximum robustness. We further concluded that in a normal environment, a healthy adult has a breathing rate of 15 bpm while lying down, a breathing rate of 18 bpm while sitting, and a breathing rate of 20 bpm while standing.

From Table 1, there are some differences in the relative errors of C-band sensing data processing results for the different postures. The relative error for standing was the lowest, followed by that for sitting, and then that for lying down was the highest. This is inversely proportional to the breathing frequency, where the higher the breathing frequency, smaller the relative error. Therefore, we concluded that the human respiratory signal is too weak; it is susceptible to interference from the surrounding environment. If the respiratory frequency is higher, there is less interference from the surrounding environment, and the relative error is lower. As for the effect of different postures on human respiratory activity, there was little change in respiratory frequency while sitting and lying down, and there was a significant change in the respiratory frequency when standing. This shows that standing has the greatest influence on respiratory activity.

## 5. Conclusions

We proposed a C-band sensing-based respiratory activity detection system that is sensitive to signal changes. The system is robust and can resist interference. This system is an improvement on the traditional breathing data filtering method, having better robustness and low relative error. This improved method was used to detect and collect data of human respiratory activity during different postures. We verified which posture has the least impact on C-band sensing data collection. For the influence of different postures on human respiratory activity, we found that standing has the greatest influence.

We only detected the respiratory signals of the human body in three postures. In the future, we want to collect more data from subjects of different age, weight, height, and sex. We plan to use this system to study the respiratory signals of human dynamic behavior, such as running and walking. As such, we will be able to draw more comprehensive conclusions.

## Figures and Tables

**Table 1:** Respiratory activity data under different postures.

Hard Threshold de-Noising	Lay	Sit	Stand
Frequency (Hz)	0.25	0.3	0.333
Respiratory Rate (bpm)	15	18	20
Relative error	<7%	<6.2%	<5.5%

## References

[1] Lu G., Wang J., Yue Y., Jing X. Study of the Ballistocardiogram signal in life detection system based on radar. Proceedings of the 29th Annual International Conference of the IEEE Engineering in Medicine and Biology Society.

[2] Jing J. (2014). Study of the Design of Radar Life Detector Based on DSP Signal Processing System. Appl. Mech. Mater..

[3] Liang X., Zhang H., Lyu T., Xu L., Cao C., Gulliver T.A. (2018). Ultra-wide band impulse radar for life detection using wavelet packetdecomposition. Phys. Commun..

[4] Liang X., Lv T., Zhang H., Gao Y., Fang G. (2018). Through-wall human being detection using UWB impulse radar. Eurasip J. Wirel. Commun. Netw..

[5] Zhen Z., Liu F. (2012). Application of Wavelet Analysis Technique in the Life Sign Detection below Solid Material. Adv. Mater. Res..

[6] Zhang Y., Jie Y. (2010). Unusual Event Detection and Prediction in Real-life Scenes. J. Shanghai Jiaotong Univ..

[7] Hu W. (2014). Research on Non-Contact Physical Signs Detection Based on Doppler Radar.

[8] Li C., Ling J., Li J., Lin J. (2010). Accurate doppler radar noncontact vital sign detection using the RELAX algorithm. IEEE Trans. Instrum. Meas..

[9] Salmi J., Molisch A.F. (2011). Propagation parameter estimation, modeling and measurements for ultrawideband MIMO radar. IEEE Trans. Antennas Propag..

[10] Ruth R., Elliot S., Ke-Yu C., Mayank G., Sidhant G., Pate S.N. Wibreathe: Estimating respiration rate using wireless signals in natural settings in the home. Proceedings of the IEEE International Conference on Pervasive Computing and Communications (PerCom).

[11] Adib F., Mao H., Kabelac Z., Katabi D., Miller R.C. Smart homes that monitor breathing and heart rate. Proceedings of the 33rd Annual ACM Conference on Human Factors in Computing Systems.

[12] Adib F., Kabelac Z., Mao H., Katabi D., Miller R.C. Demo: Real-time breath monitoring using wireless signals. Proceedings of the 20th Annual International Conference on Mobile Computing and Networking.

[13] Halperin D., Hu W., Sheth A., Wetherall D. (2010). PredicTable 802.11Packet Delivery from Wireless Channel Measurements. ACM SIGCOMM Comput. Commun. Rev..

[14] Gopalan A., Caramanis C., Shakkottai S. (2015). Wireless scheduling with partial channel state information: Large deviations and optimality. Queueing Syst..

[15] Jorswieck E.A., Boche H. (2003). Optimal Transmission with Imperfect Channel State Information at the Transmit Antenna Array. Wirel. Pers. Commun..

[16] Peng S., Liu Y., Kong Z., Lv W. (2016). Spatial Degrees of Freedom for MIMO Interference Channel with Local Channel State Information at Transmitters. Wirel. Pers. Commun..

[17] Aneja S., Sharma S. (2016). Multilevel space-time trellis coded cooperation with channel state information at transmitter. Int. J. Commun. Syst..

[18] Demeechai T., Kukieattikool P. (2017). Performance limit of AOA-based localization using MIMO-OFDM channel state information. Eurasip J. Wirel. Commun. Netw..

[19] Donoho D.L. (1995). De-noising by soft-thresholding. IEEE Trans. Inf. Theory.

[20] Donoho D.L., Johnstone I.M. (1995). Adapting to unknown smooth-ness via wavelet shrinkage. J. Am. Stat. Assoc..

[21] Donoho D.L., Johnstone I.M., Kerkyacharian G., Picard D. (1995). Wavelet shrinkage: Asymptopia?. J. R. Stat. Soc. Ser. B.

